# Demystifying the Antidepressant Mechanism of Action of Stinels, a Novel Class of Neuroplastogens: Positive Allosteric Modulators of the NMDA Receptor

**DOI:** 10.3390/ph18020157

**Published:** 2025-01-24

**Authors:** John E. Donello, Roger S. McIntyre, Donald B. Pickel, Stephen M. Stahl

**Affiliations:** 1 Gate Neurosciences, Indianapolis, IN 46202, USA; 2 Department of Psychiatry, University of Toronto, Toronto, ON M5S 1A1, Canada; 3 Metis Medical Media, Carlsbad, CA 92008, USA; 4 Department of Psychiatry and Neuroscience, University of California, Riverside, Riverside, CA 92521, USA; 5 Department of Psychiatry, University of California San Diego, La Jolla, CA 92093, USA; 6 Department of Psychiatry, University of Cambridge, Cambridge CB2 2QQ, UK; 7 California Department of State Hospitals, Sacramento, CA 95814, USA

**Keywords:** plastogens, neuroplastogens, N-methyl-D-aspartate receptor, mechanism, positive allosteric modulator, antidepressants, ketamine

## Abstract

Plastogens are a class of therapeutics that function by rapidly promoting changes in neuroplasticity. A notable example, ketamine, is receiving great attention due to its combined rapid and long-term antidepressant effects. Ketamine is an N-methyl-D-aspartate receptor (NMDAR) antagonist, and, in addition to its therapeutic activity, it is associated with psychotomimetic and dissociative side effects. Stinels—rapastinel, apimostinel, and zelquistinel—are also plastogens not only with rapid and long-term antidepressant effects but also with improved safety and tolerability profiles compared to ketamine. Previous descriptions of the mechanism by which stinels modulate NMDAR activity have been inconsistent and, at times, contradictory. The purpose of this review is to clarify the mechanism of action and contextualize stinels within a broader class of NMDAR-targeting therapeutics. In this review, we present the rationale behind targeting NMDARs for treatment-resistant depression and other psychiatric conditions, describe the various mechanisms by which NMDAR activity is regulated by different classes of therapeutics, and present evidence for the stinel mechanism. In contrast with previous descriptions of glycine-like NMDAR partial agonists, we define stinels as positive allosteric modulators of NMDAR activity with a novel regulatory binding site.

## 1. Introduction

Traditional antidepressants have focused on the modulation of monoaminergic neurotransmitter systems, such as dopamine, serotonin, and norepinephrine [1,2]. There is often a delay of up to several weeks before a clinical response is achieved [1,2]. Recently, treatments with alternative mechanisms have been successful in achieving rapid antidepressant action, with some, such as ketamine, potentiating sustained clinical improvements over time [3,4]. This class of therapeutics, called plastogens, function by rapidly promoting induced neuroplasticity [4,5,6]. Neuroplasticity is the remodeling of synapses either by trophic interactions, strengthening existing connections and synaptogenesis, or atrophic interactions, removing inactive neurons or neuronal contacts [7]. Notable among these therapeutics is ketamine, which negatively modulates N-methyl-D-aspartate receptors (NMDARs) and produces rapid and long-term antidepressant effects [2]. However, this activity also results in psychotomimetic or dissociative side effects, further classifying ketamine and molecules with similar functions as “psychoplastogens” [4]. By comparison, neuroplastogens are a class of therapeutics that typically induce neuroplasticity without inducing psychotomimetic or dissociative side effects [4,5]. Stinels—rapastinel, apimostinel, and zelquistinel—enhance synaptic structure and function with a greatly improved tolerability profile compared to NMDAR antagonists [8,9,10,11]. However, these molecules have been improperly characterized as glycine-like partial agonists of NMDAR activity, which carries the concern of potential excitotoxicity associated with other NMDAR agonists [10]. This mischaracterization, based on the results of functional assays, has been restated in reviews of glutamatergic modulators [12,13,14,15,16]. The purpose of this review is to appropriately characterize stinels as positive allosteric modulators of NMDAR activity based on recently published putative binding sites and data while highlighting the benefits of this type of modulation as it relates to neuropsychiatric disorders, including depression.

## 2. The NMDAR as a Therapeutic Target

**Figure 1:**
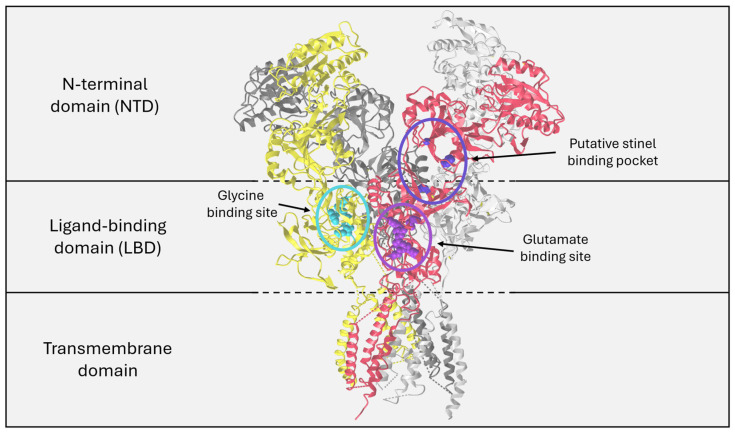
Structure and key domains of the N-methyl-D-aspartate receptor (NMDAR) and putative stinel binding site (Protein Data Bank [PDB]: 6IRH). The NMDAR is a heterotetramer of GluN subunits. GluN1 subunits are shown in yellow and light gray, and GluN2 subunits are shown in red and dark gray. Each subunit is composed of a transmembrane domain, an LBD, and an NTD. Each subunit is specific to one of its co-agonists, glycine or D-serine for GluN1 and glutamate for GluN2. The glycine binding site is highlighted in cyan, and the glutamate binding site is highlighted in purple. The putative stinel binding pocket is found at the interface of the NTD and LBD of the GluN2 subunit, highlighted in blue. Note that as a tetramer, two of each binding site are found in one NMDAR.

The architecture of NMDARs has been reviewed extensively [17,18,19]. A summary of the key domains and their functions is included in Figure 1. Briefly, NMDARs are ionotropic glutamate receptors consisting of a single transmembrane ion channel and an extracellular heterotetramer of GluN subunits. The GluN subunits may include GluN1, GluN2A-D, or GluN3A-B [19,20]. Two GluN1 subunits are obligate, and the most prevalent NMDAR subtypes are diheterotetramers of GluN1/GluN2A or GluN1/GluN2B and triheterotetramers of GluN1/GluN2A/GluN2B. Each subunit is highly conserved and is comprised of an N-terminal domain (NTD), a ligand-binding domain, and a C-terminal domain. Subunit ligand-binding domains are specific to NMDAR co-agonists, with GluN1 subunits being specific to glycine and GluN2 subunits being specific to glutamate. The NTD of NMDAR subunits has an unusual architecture relative to other ionotropic glutamate receptors that lends itself to allosteric modulation [17,19]. Different NMDAR subtypes are differentially expressed temporospatially during development and in the mature brain. The unique structural elements that comprise NMDAR subtypes allow for multiple mechanisms for modulation, making the NMDAR a valuable target for psychiatric and cognitive disorders, including depression. As subunit subtypes may help characterize NMDAR modulators, this may be relevant to their therapeutic action.

NMDARs are of particular interest in psychiatric and cognitive disorders because of their role in promoting synaptic plasticity and long-term potentiation (LTP). LTP, the basis for memory, has long been described as dependent on the activation of NMDARs [21,22,23]. The activation of the NMDAR by its co-agonists glutamate and glycine leads to calcium influx, initiating signaling cascades and resulting in increased synaptic plasticity [17,24,25]. As a result, NMDARs are considered gatekeepers facilitating long-term changes in neurological systems.

In addition to being uniquely positioned to affect long-term changes in neuroplasticity, NMDARs are also uniquely sensitive to modulation. Extensive reviews of NMDAR modulators, their structures, and putative binding sites are available [18,26]. For example, synaptic zinc is an endogenous modulator of NMDAR activity [27,28,29]. Zinc binds to an allosteric site on NMDAR subunits and inhibits channel opening and calcium influx, even in the presence of NMDAR co-agonists. The allosteric site is in the NTD of GluN subunits, which is common in noncompetitive modulators of NMDAR activity. Zinc has two binding sites on GluN2A subunits, one with high affinity and one with low affinity, and a third binding site on GluN2B subunits with lower affinity [28,30]. Because of these different properties, the modulation of NMDAR activity is altered at different concentrations of zinc, blocking long-term depression at low concentrations and inhibiting LTP at high concentrations. This modulatory profile of the NMDAR, where receptor overactivation is limited without broadly impairing excitatory signaling, may represent the goal in developing allosteric NMDAR therapeutics.

## 3. NMDAR, BDNF, and Depression

The relationship between NMDAR activity and the production of the brain-derived neurotrophic factor (BDNF) also makes the NMDAR a compelling therapeutic target. BDNF is one of the most well-characterized neurotrophins, and its activity-dependent production and subsequent activation of the neurotrophic tropomyosin receptor kinase B (TrkB) receptor are essential to changes in neuroplasticity [31,32]. Dysfunction in BDNF metabolism has been implicated in many neuropsychiatric conditions, notably depression. In fact, BDNF and TrkB have been shown to mediate the effects of many conventional antidepressants [33,34]. Stimulating BDNF production is a likely mechanism for any therapeutic whose action involves changes in neuroplasticity.

NMDAR activation leads to calcium influx, which has been shown to increase *BDNF* mRNA expression and BDNF production in neurons [35,36]. The relationship between BDNF and the NMDAR is reciprocal, as BDNF promotes the increased expression of NMDARs and the increased trafficking of NMDARs to the synaptic membrane of neurons [37]. BDNF also enhances phosphorylation of NMDAR subunits, which potentiates NMDAR activity [38]. The net result is that the NMDAR–BDNF relationship mediates changes in synaptic plasticity. These neuroplastic changes have been reported in studies with ketamine, and BDNF is thought to be necessary for its therapeutic effects [33,39]. Identifying increases in BDNF and its downstream signaling targets is a hallmark of candidate NMDAR modulators.

## 4. NMDAR Modulators

NMDARs have become a target of interest for the development of therapeutics for a number of psychiatric and cognitive disorders, including depression [12,17,40]. The NMDAR genes are highly conserved, and mutations are often associated with these conditions [17,41]. Psychiatric and cognitive disorders are associated with both excessive NMDAR activity and impaired NMDAR function, making a variety of NMDAR modulators promising therapeutics [42]. Dysfunction in NMDAR regulation has been implicated in the pathophysiology of depression [43]. Abnormalities in NMDAR genes are associated with depression, including treatment-resistant depression [44,45,46,47].

NMDAR modulators can be classified as NMDAR agonists, NMDAR antagonists, NMDAR negative allosteric modulators (NAMs), and NMDAR positive allosteric modulators (PAMs). NMDAR agonists activate the NMDAR by binding to the ligand-binding domain [19,20]. NMDAR agonists either bind to the orthosteric glutamate site, the same site where the co-agonist glutamate binds, or to the orthosteric glycine site, or the same site where the co-agonists glycine and D-serine bind [2,19,20]. Some NMDAR antagonists block the orthosteric binding of the endogenous NMDAR agonists glycine, D-serine, or glutamate by occluding the orthosteric ligand-binding domains, thereby inhibiting NMDAR activity [19,20]. Other NMDAR antagonists are allosteric modulators that bind noncompetitively at NMDAR sites other than the orthosteric ligand-binding domain, often in the NTD, and modulate NMDAR activity by altering the receptor’s sensitivity to its agonists [19,20,48].

Thus, there are multiple molecular mechanisms by which antagonists can negatively modulate receptor activity, including competitive antagonism, uncompetitive antagonism, and noncompetitive antagonism [49]. Competitive antagonists directly block the orthosteric ligand-binding domains of the target receptor. Uncompetitive antagonists do not bind to the orthosteric site of their targets, but, importantly, they bind only to activated receptors, requiring the presence of a ligand binding to the orthosteric site to function. These uncompetitive allosteric antagonists typically have minimal effects on receptor activity at low levels of ligand, but they have greater inhibitory activity at high levels of ligand [50]. Uncompetitive antagonists occlude structures like ion channels once they are opened following receptor activation by its ligands. Noncompetitive antagonists, or NAMs, negatively modulate the reactivity of a receptor without binding to its orthosteric site. Allosteric modulators frequently offer a measure of receptor subtype specificity, generally do not act at orthosteric ligand sites, and do not activate the receptor in the absence of its ligands [48,51,52]. In contrast to NAMs, which reduce receptor reactivity, there are PAMs, which potentiate receptor reactivity.

Many classes of therapeutics that are thought to function by modulating NMDAR activity have been developed. Examples are summarized in Table 1. Most NMDAR antagonists are uncompetitive antagonists binding to the open ion channel conformation of activated NMDARs. These include dextromethorphan, lanicemine, riluzole, CERC-301, memantine, methadone, esmethadone, and nitrous oxide, as well as ketamine and its enantiomers, esketamine and arketamine [53,54,55,56,57,58,59,60,61,62]. AV-101 is an orthosteric glycine site–specific NMDAR antagonist being developed for use in depression and other neurological disorders [63]. Sarcosine is an orthosteric glycine site-dependent NMDAR agonist, and D-cycloserine is an orthosteric glycine site NMDAR partial agonist [64,65]. Several NMDAR NAMs, such as ifenprodil, traxoprodil (CP-101606), Ro 25-6891, BMS-986163, YY-23, and onfasprodil (MIJ821), are commonly called antagonists but do not bind to the orthosteric glutamate site or the orthosteric glycine site and instead have allosteric modulatory activity specific to GluN2B subunits [26,66,67].

## 5. Stinels Are NMDAR PAMs

**Figure 2:**
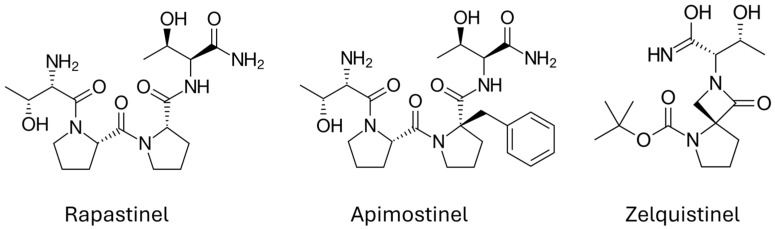
Chemical structures of the stinels.

Stinels represent different generations of NMDAR modulators with different pharmacologic properties, which are thought to function through a similar mechanism. Their chemical structures are shown in Figure 2. The development of rapastinel was the result of refining an antibody that was initially created to investigate the molecular mechanisms underlying neuroplasticity [68]. When isolating regions of the antibody’s hypervariable region, the amidated tetrapeptide was found to have the highest binding affinity for the NMDAR. The potency for rapastinel at the NMDAR was measured using a primary cortical neuron NMDA-induced calcium flux assay, where rapastinel maximally enhanced calcium flux at a concentration of 100 nM [69]. Apimostinel is also an amidated tetrapeptide, but it has been structurally modified with the addition of a benzyl group, improving its bioavailability and binding affinity, giving it increased potency relative to rapastinel [70]. Zelquistinel diverges from its predecessors with no peptide backbone. It has improved potency relative to rapastinel, and it is also orally stable. In a calcium influx assay, zelquistinel maximally potentiated NMDAR activity at a five-fold lower concentration [69,71]. The differences and similarities of these three molecules are summarized in Table 2.

Rapastinel was developed from an antibody that was shown to act like an NMDAR glycine site partial agonist, so it and the other stinels were characterized with that mechanism in mind. However, the previous descriptions of stinels were based on functional assays [68]. New studies show that stinels are not glycine site partial agonists and instead modulate NMDAR activity independent of the glycine site [69,71]. Radioligand displacement assays found that neither rapastinel nor zelquistinel binds to the glycine site or any other known modulatory binding site. Both stinels potentiated NMDAR reactivity to its ligand glutamate in the presence of MDL 105,519, a known glycine site competitive antagonist. Notably, rapastinel and zelquistinel required the presence of glutamate to potentiate NMDAR reactivity [69,71]. Altogether, this shows that stinels—rapastinel, apimostinel, and zelquistinel—potentiate NMDAR reactivity only in the presence of glutamate and its co-agonists glycine and D-serine and should be considered NMDAR PAMs [69,71].

Specifically, rapastinel, apimostinel, and zelquistinel are NMDAR PAMs that act at a novel binding site in the NTD [69,71,72]. A novel binding site in the NTD of GluN2 subunits for rapastinel is predicted by in silico modeling [69]. The putative binding site is shown in Figure 1 and can be seen as distinct from the glycine binding site. Point mutagenesis of this binding site resulted in no change in activation of the NMDAR by its ligands glutamate and D-serine, but it completely ablated the rapastinel-mediated potentiation of NMDAR signaling [69]. Site-directed mutagenesis of the same binding pocket demonstrated that zelquistinel binds to the same site [72]. Some data regarding stinel selectivity for specific GluN2 subtypes are available, but how this mediates the effects of stinels is unclear and should be the subject of further study [72].

NMDAR-mediated calcium influx is associated with synaptogenesis and synaptic plasticity. Stinels may also play a role in regulating calcium influx through the ion channel of activated NMDARs. Rapastinel and zelquistinel enhanced the gate conductance of NMDARs [72,73]. The C-terminal tail of the GluN1 subunit of the NMDAR contains a binding site for calcium-bound calmodulin that inhibits calcium conductance following receptor activation [74]. Stinels may reduce the affinity of this internal inactivation site, possibly due to a unique conformational change in NMDAR structure, leading to potentiated ion transfer.

## 6. Convergent Antidepressant Effects of Ketamine and Stinels

The success of ketamine as both a rapid antidepressant and an initiator of long-term improvements in synaptic function has drawn attention to NMDAR modulation as a potential therapeutic target. The rapid antidepressant effects of ketamine may be related to BDNF–TrkB signaling [33,39]. Notably, ketamine has been shown to increase BDNF protein expression, but not gene expression, perhaps due to the desuppression of eukaryotic elongation factor 2. BDNF binds to the TrkB receptor with high affinity. A critical role for this interaction has been established in synaptic plasticity. Ketamine has also been shown to stimulate other downstream signaling pathways, such as extracellular-regulated protein kinase (ERK), mammalian target of rapamycin (mTOR), and glycogen synthase kinase 3, all of which have been implicated in synaptogenesis [33,39,75,76]. Indeed, ketamine treatment leads to changes in neuroplasticity mediated by enhanced synapse formation [77,78].

**Figure 3:**
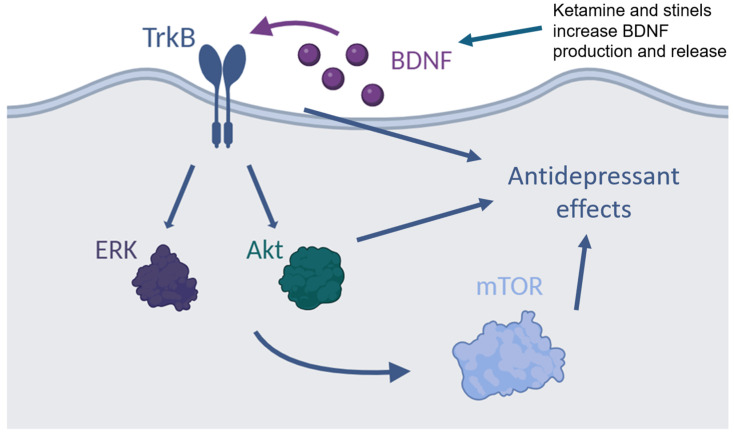
Molecular mechanisms of the convergent antidepressant effects of ketamine and stinels. The antidepressant effects of both ketamine and stinels are associated with changes in molecular signaling pathways. Increased brain-derived neurotrophic factor (BDNF) production and release have been found with both NMDAR modulators. BDNF binds to the tropomyosin receptor kinase B (TrkB) receptor, activating intracellular signaling cascades including extracellular-regulated protein kinase (ERK) and tyrosine kinase B (Akt). Finally, elevation in mammalian target of rapamycin (mTOR) signaling mediates antidepressant effects.

Like ketamine, rapastinel and zelquistinel produce rapid and sustained antidepressant effects in animal models and the preliminary studies of depressed patients [69,71]. The antidepressant effects of rapastinel have been associated with the activation of the ERK and mTOR signaling pathways [79,80,81]. Data have shown that the antidepressant effects of rapastinel are dependent on BDNF release [82]. Rapastinel can induce neuroplastic changes, with a single dose enhancing LTP and increasing dendritic spine density [9]. Similarly, zelquistinel enhanced LTP for up to two weeks following a single dose [71]. Figure 3 summarizes the convergent molecular mechanisms associated with the antidepressant effects of ketamine and stinels.

Interestingly, these similarities in convergent antidepressant effects are the result of contrasting modulatory activity. Contrasting regulatory mechanisms of NMDAR activity by ketamine and stinels—an antagonist and a PAM, respectively—surprisingly lead to similar downstream effects resulting from the convergent disinhibition of pyramidal neurons [83]. Notably, there are differences in how the signaling molecules and pathways are affected by each agent. Ketamine elicits an acute release of glutamate, while rapastinel does not [79]. That is, despite the fact that ketamine blocks NMDARs, the downstream result is the enhanced release of glutamate due to this disinhibition [2]. The effects of ketamine on the downstream processes associated with enhanced plasticity may therefore be indirect, mediated by the NMDAR inhibition–dependent pathways, with the release of glutamate itself and the stimulation of α-amino-3-hydroxy-5-methyl-4-isoxazolepropionic acid receptors (AMPARs). On the other hand, stinels directly potentiate NMDAR activity, possibly on postsynaptic neurons. Both lead to postsynaptic changes in intracellular signaling pathways driving neuroplasticity. There are some evident differences in the dynamic modulation of the molecular pathways mediating antidepressant effects. When administered intravenously, ketamine did not elicit observable changes in the activated state of some signaling proteins, including protein kinase B, mTOR, and extracellular signal-related kinases, yet rapastinel did [79]. This may be evidence of temporal differences in how these pathways are stimulated.

**Figure 4:**
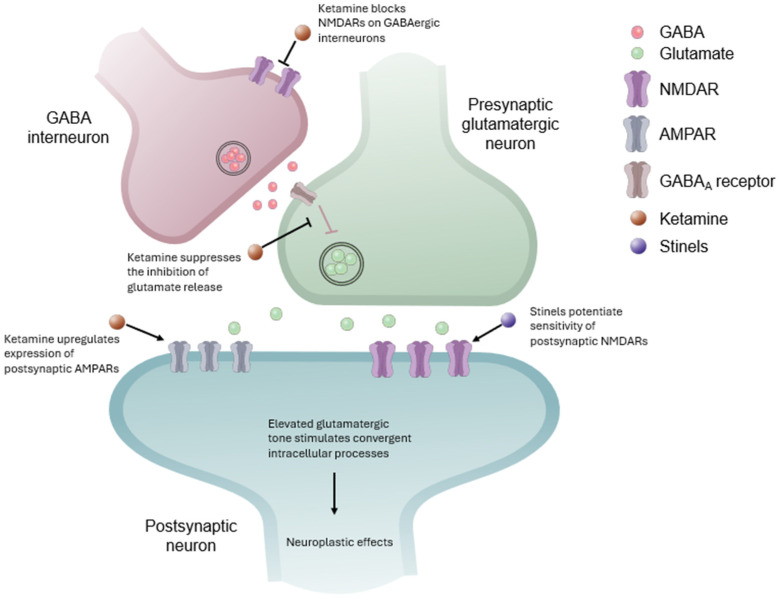
Putative divergent mechanisms of ketamine and stinels. The antidepressant effects of ketamine are most often associated with two mechanisms. The disinhibition mechanism is NMDAR dependent and involves blockade of NMDARs on GABAergic interneurons, leading to suppression of γ-aminobutyric acid (GABA)-mediated inhibition of glutamate release. An NMDAR-independent mechanism is supported by studies of ketamine metabolites, which involved upregulation of the expression of postsynaptic α-amino-3-hydroxy-5-methyl-4-isoxazolepropionic acid receptors (AMPARs), leading to increased sensitivity to this elevated glutamatergic tone. Stinels potentiate sensitivity of postsynaptic NMDARs. These different mechanisms converge in stimulating postsynaptic intracellular signaling mechanisms to produce neuroplastic effects. GABA_A_, γ-aminobutyric acid type A.

One possible difference that requires further investigation is that ketamine and stinels may preferentially act at different NMDARs on different neuronal populations [79]. Figure 4 depicts the putative mechanisms of ketamine and the stinels. The antidepressant effects of ketamine are predominantly attributed to the antagonism of NMDARs. Often called the disinhibition hypothesis, the putative selective blockade of GluN2B-containing NMDARs on GABAergic interneurons leads to a suppression of the inhibition of glutamate release by presynaptic glutamatergic neurons [84,85]. Recent studies using ketamine metabolites, which do not bind to NMDARs, suggest that the antidepressant activity of ketamine may be independent of this mechanism [86,87]. These studies show that ketamine and its metabolites are associated with increased postsynaptic expression of AMPARs [86,87]. Indeed, it has been shown previously that AMPAR activity is essential for the antidepressant effects of both ketamine and rapastinel [81]. It is possible that these distinct mechanisms may have synergistic effects, explaining why other NMDAR antagonists do not have the same antidepressant effects [88]. In contrast to ketamine, stinels directly potentiate NMDARs. It has been shown that NMDAR PAMs may have distinct mechanisms, and some may selectively potentiate NMDARs on excitatory neurons [89]. The exact nature of NMDAR selectivity is the subject of ongoing research and likely involves the specificity of NMDAR diheteromers and triheteromers, as well as glutamate, glycine, and D-serine availability [48,89,90]. Reconciling the divergent pharmacology of stinels with NMDAR antagonists, such as ketamine, and the convergent antidepressant effects may also explain the improved psychotomimetic or dissociative side effect profile of stinels at therapeutic doses, which is also observed with some ketamine metabolites [86]. Notably, the antidepressant effects of ketamine are ablated by orthosteric NMDAR antagonists [91]. While a dependence on NMDAR activation may seem contradictory to the mechanism of an NMDAR antagonist, the metabolite hypothesis suggests a more direct relationship between glutamatergic signaling and the antidepressant effects of ketamine.

## 7. Current Challenges and Future Efforts

Ongoing research highlights the unknown elements of NMDAR-dependent activation by stinels for future study. Recently, a classification system of PAMs was introduced, in which type I PAMs enhance the activity of a receptor without altering the half-maximal effective concentration (EC_50_) of its ligand, while type II PAMs lower the EC_50_ of a ligand [90]. Stinels may have characteristics of both classes. Stinels are proposed to bind to a novel regulatory site in the NTD of GluN2 subunits. Rapastinel has been shown to have affinity for each of the four GluN2 subtypes, but subtype specificity should be the subject of further study [69]. NMDARs with specific subunits are differentially expressed by different neuronal subpopulations, and these expression profiles may further have selectivity for specific subtypes. There is some evidence that stinels interact with NMDARs on specific neuron subpopulations, but characterizing subtype specificity and how that determines initial targets is the subject of ongoing studies [92].

In addition to the acute side effects, there are some concerns with long-term safety with the use of NMDAR antagonists. Thus, neurotoxicity and organ toxicity stemming from the first observation of lesions following a single dose of ketamine in rats in 1989 have been reported [93]. Because PAMs potentiate receptor activity only in the presence of its ligand, there is no evidence that PAMs carry similar issues. Recently, the preclinical evaluations of apimostinel and zelquistinel have shown that they elicit rapid antidepressant effects in animal models and induce metaplastic changes potentiating sustained antidepressant effects. Zelquistinel was well tolerated in phase 2 clinical trials. Currently, these stinels are in phase 2 clinical trials for efficacy in treating major depressive disorder.

## 8. Conclusions

The recent success of NMDAR antagonists, notably ketamine, in the treatment of depression has turned attention toward this receptor for the development of novel therapeutics. Ketamine and similar agents are notable for having both rapid and sustained antidepressant effects. The long-term effects of these agents are thought to be mediated by enhanced neuroplasticity, classifying them as plastogens. However, NMDAR antagonists often have psychotomimetic and dissociative side effects, further classifying them as psychoplastogens, and there are concerns about neurotoxicity and organ toxicity with long-term treatment. Stinels—rapastinel, apimostinel, and zelquistinel—are PAMs that potentiate NMDAR activity independently of the orthosteric ligand-binding domain. The stinels have both rapid and sustained antidepressant activity in animal models and preliminary clinical testing, like other plastogens, but have improved safety and tolerability, often without the accompanying psychotomimetic and dissociative side effects associated with ketamine, further classifying them as neuroplastogens. Stinels offer a novel approach to the rapid, durable, and safe enhancement of synaptic function, providing therapeutic potential for depression and other psychiatric or cognitive disorders, and they warrant further investigation and research.

## Figures and Tables

**Table 1:** Summary of NMDAR modulators.

Mechanism	NMDAR Modulator
	**NMDAR antagonists**
Uncompetitive antagonism	Ketamine
	Esketamine
	Arketamine
	Dextromethorphan
	Lanicemine
	Riluzole
	CERC-301
	Memantine
	Methadone
	Esmethadone
	Nitrous oxide
Glycine site competitive antagonism	AV-101
	**NMDAR agonists**
Glycine site NMDAR agonist	Sarcosine
Glycine site NMDAR partial agonist	D-cycloserine
	**NMDAR negative allosteric modulator** **s (NAMs)**
Noncompetitive antagonism	Zinc Ifenprodil
	Traxoprodil (CP-101606)
	Ro 25-6891
	BMS-986163
	YY-23
	Onfasprodil (MIJ821)
	**NMDAR positive allosteric modulators (PAMs)**
Noncompetitive agonism	GNE-6901
	GNE-8324
	Rapastinel (GLYX-13)
	Apimostinel (NRX-1074)
	Zelquistinel (AGN-241751)

**Table 2:** Summary of stinel characteristics.

Stinel *	Stinel Generation	Chemistry	NMDAR Potency ^†^	Oral Availability
Rapastinel	First	Peptide	-	No
Apimostinel	Second	Peptide	10–30×	No
Zelquistinel	Third	Small molecule	3–10×	Yes

* The stinels included in this manuscript are the most well characterized. ^†^ Potency shown is at the NMDAR relative to rapastinel.

## References

[1] Harmer C.J., Duman R.S., Cowen P.J. (2017). How do antidepressants work? New perspectives for refining future treatment approaches. Lancet Psychiatry.

[2] Stahl S.M. (2021). Stahl’s Essential Psychopharmacology: Neuroscientific Basis and Practical Applications.

[3] Newport D.J., Carpenter L.L., McDonald W.M., Potash J.B., Tohen M., Nemeroff C.B., The APA Council of Research Task Force on Novel Biomarkers and Treatments (2015). Ketamine and other NMDA antagonists: Early clinical trials and possible mechanisms in depression. Am. J. Psychiatry.

[4] Cooper T., Seigler M.D., Stahl S. (2023). Rapid onset brain plasticity at novel pharmacologic targets hypothetically drives innovations for rapid onset antidepressant actions. J. Psychopharmacol..

[5] Ly C., Greb A.C., Cameron L.P., Wong J.M., Barragan E.V., Wilson P.C., Burbach K.F., Soltanzadeh Zarandi S., Sood A., Paddy M.R. (2018). Psychedelics promote structural and functional neural plasticity. Cell Rep..

[6] Olson D.E. (2018). Psychoplastogens: A promising class of plasticity-promoting neurotherapeutics. J. Exp. Neurosci..

[7] Castren E., Antila H. (2017). Neuronal plasticity and neurotrophic factors in drug responses. Mol. Psychiatry.

[8] Burgdorf J., Zhang X.L., Nicholson K.L., Balster R.L., Leander J.D., Stanton P.K., Gross A.L., Kroes R.A., Moskal J.R. (2013). GLYX-13, a NMDA receptor glycine-site functional partial agonist, induces antidepressant-like effects without ketamine-like side effects. Neuropsychopharmacology.

[9] Burgdorf J., Zhang X.L., Weiss C., Gross A., Boikess S.R., Kroes R.A., Khan M.A., Burch R.M., Rex C.S., Disterhoft J.F. (2015). The long-lasting antidepressant effects of rapastinel (GLYX-13) are associated with a metaplasticity process in the medial prefrontal cortex and hippocampus. Neuroscience.

[10] Moskal J.R., Burch R., Burgdorf J.S., Kroes R.A., Stanton P.K., Disterhoft J.F., Leander J.D. (2014). GLYX-13, an NMDA receptor glycine site functional partial agonist enhances cognition and produces antidepressant effects without the psychotomimetic side effects of NMDA receptor antagonists. Expert Opin. Investig. Drugs.

[11] Pothula S., Liu R.J., Wu M., Sliby A.N., Picciotto M.R., Banerjee P., Duman R.S. (2021). Positive modulation of NMDA receptors by AGN-241751 exerts rapid antidepressant-like effects via excitatory neurons. Neuropsychopharmacology.

[12] Henter I.D., de Sousa R.T., Zarate C.A. (2018). Glutamatergic modulators in depression. Harv. Rev. Psychiatry.

[13] Wilkinson S.T., Sanacora G. (2019). A new generation of antidepressants: An update on the pharmaceutical pipeline for novel and rapid-acting therapeutics in mood disorders based on glutamate/GABA neurotransmitter systems. Drug Discov. Today.

[14] Henter I.D., Park L.T., Zarate C.A. (2021). Novel glutamatergic modulators for the treatment of mood disorders: Current status. CNS Drugs.

[15] Chrobak A.A., Siwek M. (2024). Drugs with glutamate-based mechanisms of action in psychiatry. Pharmacol. Rep..

[16] Haring R., Stanton P.K., Scheideler M.A., Moskal J.R. (1991). Glycine-like modulation of N-methyl-D-aspartate receptors by a monoclonal antibody that enhances long-term potentiation. J. Neurochem..

[17] Stroebel D., Paoletti P. (2021). Architecture and function of NMDA receptors: An evolutionary perspective. J. Physiol..

[18] Hansen K.B., Yi F., Perszyk R.E., Furukawa H., Wollmuth L.P., Gibb A.J., Traynelis S.F. (2018). Structure, function, and allosteric modulation of NMDA receptors. J. Gen. Physiol..

[19] Vyklicky V., Korinek M., Smejkalova T., Balik A., Krausova B., Kaniakova M., Lichnerova K., Cerny J., Krusek J., Dittert I. (2014). Structure, function, and pharmacology of NMDA receptor channels. Physiol. Res..

[20] Glasgow N.G., Siegler Retchless B., Johnson J.W. (2015). Molecular bases of NMDA receptor subtype-dependent properties. J. Physiol..

[21] Lynch M.A. (2004). Long-term potentiation and memory. Physiol. Rev..

[22] Nicoll R.A. (2017). A brief history of long-term potentiation. Neuron.

[23] Volianskis A., France G., Jensen M.S., Bortolotto Z.A., Jane D.E., Collingridge G.L. (2015). Long-term potentiation and the role of N-methyl-D-aspartate receptors. Brain Res..

[24] Lüscher C., Malenka R.C. (2012). NMDA receptor-dependent long-term potentiation and long-term depression (LTP/LTD). Cold Spring Harb. Perspect. Biol..

[25] Pittenger C., Sanacora G., Krystal J.H. (2007). The NMDA receptor as a therapeutic target in major depressive disorder. CNS Neurol. Disord. Drug Targets.

[26] Hanson J.E., Yuan H., Perszyk R.E., Banke T.G., Xing H., Tsai M.C., Menniti F.S., Traynelis S.F. (2024). Therapeutic potential of N-methyl-D-aspartate receptor modulators in psychiatry. Neuropsychopharmacology.

[27] Paoletti P., Ascher P., Neyton J. (1997). High-affinity zinc inhibition of NMDA NR1-NR2A receptors. J. Neurosci..

[28] Petrilli M.A., Kranz T.M., Kleinhaus K., Joe P., Getz M., Johnson P., Chao M.V., Malaspina D. (2017). The emerging role for zinc in depression and psychosis. Front. Pharmacol..

[29] Anderson C.T., Radford R.J., Zastrow M.L., Zhang D.Y., Apfel U.P., Lippard S.J., Tzounopoulos T. (2015). Modulation of extrasynaptic NMDA receptors by synaptic and tonic zinc. Proc. Natl. Acad. Sci. USA.

[30] Izumi Y., Auberson Y.P., Zorumski C.F. (2006). Zinc modulates bidirectional hippocampal plasticity by effects on NMDA receptors. J. Neurosci..

[31] Leal G., Comprido D., Duarte C.B. (2014). BDNF-induced local protein synthesis and synaptic plasticity. Neuropharmacology.

[32] Colucci-D’Amato L., Speranza L., Volpicelli F. (2020). Neurotrophic factor BDNF, physiological functions and therapeutic potential in depression, neurodegeneration and brain cancer. Int. J. Mol. Sci..

[33] Bjorkholm C., Monteggia L.M. (2016). BDNF—A key transducer of antidepressant effects. Neuropharmacology.

[34] Casarotto P., Umemori J., Castren E. (2022). BDNF receptor TrkB as the mediator of the antidepressant drug action. Front. Mol. Neurosci..

[35] Gwag B.J., Springer J.E. (1993). Activation of NMDA receptors increases brain-derived neurotrophic factor (BDNF) mRNA expression in the hippocampal formation. Neuroreport.

[36] Finkbeiner S. (2000). Calcium regulation of the brain-derived neurotrophic factor gene. Cell. Mol. Life. Sci..

[37] Caldeira M.V., Melo C.V., Pereira D.B., Carvalho R.F., Carvalho A.L., Duarte C.B. (2007). BDNF regulates the expression and traffic of NMDA receptors in cultured hippocampal neurons. Mol. Cell. Neurosci..

[38] Suen P.C., Wu K., Levine E.S., Mount H.T., Xu J.L., Lin S.Y., Black I.B. (1997). Brain-derived neurotrophic factor rapidly enhances phosphorylation of the postsynaptic N-methyl-D-aspartate receptor subunit 1. Proc. Natl. Acad. Sci. USA.

[39] Zanos P., Gould T.D. (2018). Mechanisms of ketamine action as an antidepressant. Mol. Psychiatry.

[40] Adell A. (2020). Brain NMDA receptors in schizophrenia and depression. Biomolecules.

[41] Amin J.B., Moody G.R., Wollmuth L.P. (2021). From bedside-to-bench: What disease-associated variants are teaching us about the NMDA receptor. J. Physiol..

[42] McIntyre R.S., Rosenblat J.D., Rodrigues N.B., Lipsitz O., Chen-Li D., Lee J.G., Nasri F., Subramaniapillai M., Kratiuk K., Wang A. (2021). The effect of intravenous ketamine on cognitive functions in adults with treatment-resistant major depressive or bipolar disorders: Results from the Canadian Rapid Treatment Center of Excellence (CRTCE). Psychiatry Res..

[43] Marsden W.N. (2011). Stressor-induced NMDAR dysfunction as a unifying hypothesis for the aetiology, pathogenesis and comorbidity of clinical depression. Med. Hypotheses.

[44] Kaut O., Schmitt I., Hofmann A., Hoffmann P., Schlaepfer T.E., Wullner U., Hurlemann R. (2015). Aberrant NMDA receptor DNA methylation detected by epigenome-wide analysis of hippocampus and prefrontal cortex in major depression. Eur. Arch. Psychiatry Clin. Neurosci..

[45] Niciu M.J., Ionescu D.F., Richards E.M., Zarate C.A. (2014). Glutamate and its receptors in the pathophysiology and treatment of major depressive disorder. J. Neural Transm..

[46] Zhang C., Li Z., Wu Z., Chen J., Wang Z., Peng D., Hong W., Yuan C., Wang Z., Yu S. (2014). A study of N-methyl-D-aspartate receptor gene (GRIN2B) variants as predictors of treatment-resistant major depression. Psychopharmacology.

[47] McIntyre R.S., Alsuwaidan M., Baune B.T., Berk M., Demyttenaere K., Goldberg J.F., Gorwood P., Ho R., Kasper S., Kennedy S.H. (2023). Treatment-resistant depression: Definition, prevalence, detection, management, and investigational interventions. World Psychiatry.

[48] Geoffroy C., Paoletti P., Mony L. (2022). Positive allosteric modulation of NMDA receptors: Mechanisms, physiological impact and therapeutic potential. J. Physiol..

[49] Ogden K.K., Traynelis S.F. (2011). New advances in NMDA receptor pharmacology. Trends Pharmacol. Sci..

[50] Lipton S.A. (2004). Failures and successes of NMDA receptor antagonists: Molecular basis for the use of open-channel blockers like memantine in the treatment of acute and chronic neurologic insults. NeuroRx.

[51] Abdel-Magid A.F. (2015). Allosteric modulators: An emerging concept in drug discovery. ACS Med. Chem. Lett..

[52] Cao A.M., Quast R.B., Fatemi F., Rondard P., Pin J.P., Margeat E. (2021). Allosteric modulators enhance agonist efficacy by increasing the residence time of a GPCR in the active state. Nat. Commun..

[53] Ebert B., Mikkelsen S., Thorkildsen C., Borgbjerg F.M. (1997). Norketamine, the main metabolite of ketamine, is a non-competitive NMDA receptor antagonist in the rat cortex and spinal cord. Eur. J. Pharmacol..

[54] Zhang Y., Ye F., Zhang T., Lv S., Zhou L., Du D., Lin H., Guo F., Luo C., Zhu S. (2021). Author correction: Structural basis of ketamine action on human NMDA receptors. Nature.

[55] Zorumski C.F., Izumi Y., Mennerick S. (2016). Ketamine: NMDA receptors and beyond. J. Neurosci..

[56] Taylor C.P., Traynelis S.F., Siffert J., Pope L.E., Matsumoto R.R. (2016). Pharmacology of dextromethorphan: Relevance to dextromethorphan/quinidine (Nuedexta^®^) clinical use. Pharmacol. Ther..

[57] Garner R., Gopalakrishnan S., McCauley J.A., Bednar R.A., Gaul S.L., Mosser S.D., Kiss L., Lynch J.J., Patel S., Fandozzi C. (2015). Preclinical pharmacology and pharmacokinetics of CERC-301, a GluN2B-selective N-methyl-D-aspartate receptor antagonist. Pharmacol. Res. Perspect..

[58] Bettini E., Stahl S.M., De Martin S., Mattarei A., Sgrignani J., Carignani C., Nola S., Locatelli P., Pappagallo M., Inturrisi C.E. (2022). Pharmacological comparative characterization of REL-1017 (esmethadone-HCl) and other NMDAR channel blockers in human heterodimeric N-methyl-D-aspartate receptors. Pharmaceuticals.

[59] Song X., Jensen M.O., Jogini V., Stein R.A., Lee C.H., McHaourab H.S., Shaw D.E., Gouaux E. (2018). Mechanism of NMDA receptor channel block by MK-801 and memantine. Nature.

[60] Jevtović-Todorović V., Todorovć S.M., Mennerick S., Powell S., Dikranian K., Benshoff N., Zorumski C.F., Olney J.W. (1998). Nitrous oxide (laughing gas) is an NMDA antagonist, neuroprotectant and neurotoxin. Nat. Med..

[61] Sanacora G., Smith M.A., Pathak S., Su H.L., Boeijinga P.H., McCarthy D.J., Quirk M.C. (2014). Lanicemine: A low-trapping NMDA channel blocker produces sustained antidepressant efficacy with minimal psychotomimetic adverse effects. Mol. Psychiatry.

[62] Doble A. (1996). The pharmacology and mechanism of action of riluzole. Neurology.

[63] Bourque M., Gregoire L., Patel W., Dickens D., Snodgrass R., Di Paolo T. (2022). AV-101, a pro-drug antagonist at the NMDA receptor glycine site, reduces L-dopa induced dyskinesias in MPTP monkeys. Cells.

[64] Lee M.Y., Lin Y.R., Tu Y.S., Tseng Y.J., Chan M.H., Chen H.H. (2017). Effects of sarcosine and N, N-dimethylglycine on NMDA receptor-mediated excitatory field potentials. J. Biomed. Sci..

[65] Schade S., Paulus W. (2016). D-cycloserine in neuropsychiatric diseases: A systematic review. Int. J. Neuropsychopharmacol..

[66] Gomez-Mancilla B., Levy J.A., Ganesan S., Faller T., Issachar G., Peremen Z., Laufer O., Shani-Hershkovich R., Biliouris K., Walker E. (2023). MIJ821 (onfasprodil) in healthy volunteers: First-in-human, randomized, placebo-controlled study (single ascending dose and repeated intravenous dose). Clin. Transl. Sci..

[67] Marcin L.R., Warrier J., Thangathirupathy S., Shi J., Karageorge G.N., Pearce B.C., Ng A., Park H., Kempson J., Li J. (2018). BMS-986163, a negative allosteric modulator of GluN2B with potential utility in major depressive disorder. ACS Med. Chem. Lett..

[68] Moskal J.R., Burgdorf J.S., Stanton P.K., Kroes R.A., Disterhoft J.F., Burch R.M., Khan M.A. (2017). The development of rapastinel (formerly GLYX-13); a rapid acting and long lasting antidepressant. Curr. Neuropharmacol..

[69] Donello J.E., Banerjee P., Li Y.X., Guo Y.X., Yoshitake T., Zhang X.L., Miry O., Kehr J., Stanton P.K., Gross A.L. (2019). Positive N-methyl-D-aspartate receptor modulation by rapastinel promotes rapid and sustained antidepressant-like effects. Int. J. Neuropsychopharmacol..

[70] Fasipe O.J. (2019). The emergence of new antidepressants for clinical use: Agomelatine paradox versus other novel agents. IBRO Rep..

[71] Burgdorf J.S., Zhang X.L., Stanton P.K., Moskal J.R., Donello J.E. (2022). Zelquistinel is an orally bioavailable novel NMDA receptor allosteric modulator that exhibits rapid and sustained antidepressant-like effects. Int. J. Neuropsychopharmacol..

[72] Zhang X.L., Li Y.X., Berglund N., Burgdorf J.S., Donello J.E., Moskal J.R., Stanton P.K. (2024). Zelquistinel acts at an extracellular binding domain to modulate intracellular calcium inactivation of N-methyl-d-aspartate receptors. Neuropharmacology.

[73] Zhang X.L., Berglund N.A., Burgdorf J.S., Donello J.E., Moskal J.R., Stanton P.K. (2022). Extracellular application of the N-methyl-D-aspartate receptor allosteric modulator rapastinel acts remotely to regulate Ca2+ inactivation at an intracellular locus. Neuroreport.

[74] Bhatia N.K., Carrillo E., Durham R.J., Berka V., Jayaraman V. (2020). Allosteric changes in the NMDA receptor associated with calcium-dependent inactivation. Biophys. J..

[75] Li N., Lee B., Liu R.J., Banasr M., Dwyer J.M., Iwata M., Li X.Y., Aghajanian G., Duman R.S. (2010). mTOR-dependent synapse formation underlies the rapid antidepressant effects of NMDA antagonists. Science.

[76] Ma Z., Zang T., Birnbaum S.G., Wang Z., Johnson J.E., Zhang C.L., Parada L.F. (2017). TrkB dependent adult hippocampal progenitor differentiation mediates sustained ketamine antidepressant response. Nat. Commun..

[77] Duman R.S. (2014). Pathophysiology of depression and innovative treatments: Remodeling glutamatergic synaptic connections. Dialogues Clin. Neurosci..

[78] Brown K.A., Gould T.D. (2024). Targeting metaplasticity mechanisms to promote sustained antidepressant actions. Mol. Psychiatry.

[79] Lee B., Pothula S., Duman R.S. (2020). NMDAR modulators as rapid antidepressants: Converging and distinct signaling mechanisms. Integr. Clin. Med..

[80] Shen M., Lv D., Liu X., Wang C. (2022). ERK/mTOR signaling may underlying the antidepressant actions of rapastinel in mice. Transl. Psychiatry.

[81] Lepack A.E., Bang E., Lee B., Dwyer J.M., Duman R.S. (2016). Fast-acting antidepressants rapidly stimulate ERK signaling and BDNF release in primary neuronal cultures. Neuropharmacology.

[82] Kato T., Fogaca M.V., Deyama S., Li X.Y., Fukumoto K., Duman R.S. (2018). BDNF release and signaling are required for the antidepressant actions of GLYX-13. Mol. Psychiatry.

[83] Widman A.J., McMahon L.L. (2018). Disinhibition of CA1 pyramidal cells by low-dose ketamine and other antagonists with rapid antidepressant efficacy. Proc. Natl. Acad. Sci. USA.

[84] Borsellino P., Krider R.I., Chea D., Grinnell R., Vida T.A. (2023). Ketamine and the disinhibition hypothesis: Neurotrophic factor-mediated treatment of depression. Pharmaceuticals.

[85] Kim J., Suzuki K., Kavalali E., Monteggia L. (2023). Bridging rapid and sustained antidepressant effects of ketamine. Trends Mol. Med..

[86] Zanos P., Moaddel R., Morris P.J., Georgiou P., Fischell J., Elmer G.I., Alkondon M., Yuan P., Pribut H.J., Singh N.S. (2016). NMDAR inhibition-independent antidepressant actions of ketamine metabolites. Nature.

[87] Zanos P., Moaddel R., Morris P.J., Riggs L.M., Highland J.N., Georgiou P., Pereira E.F.R., Albuquerque E.X., Thomas C.J., Zarate C.A. (2018). Ketamine and ketamine metabolite pharmacology: Insights into therapeutic mechanisms. Pharmacol. Rev..

[88] Du J., Machado-Vieira R., Maeng S., Martinowich K., Manji H.K., Zarate C.A. (2006). Enhancing AMPA to NMDA throughput as a convergent mechanism for antidepressant action. Drug Discov. Today Ther. Strateg..

[89] Hackos D.H., Lupardus P.J., Grand T., Chen Y., Wang T.M., Reynen P., Gustafson A., Wallweber H.J., Volgraf M., Sellers B.D. (2016). Positive allosteric modulators of GluN2A-containing NMDARs with distinct modes of action and impacts on circuit function. Neuron.

[90] Hackos D.H., Hanson J.E. (2017). Diverse modes of NMDA receptor positive allosteric modulation: Mechanisms and consequences. Neuropharmacology.

[91] Zanos P., Brown K.A., Georgiou P., Yuan P., Zarate C.A., Thompson S.M., Gould T.D. (2023). NMDA receptor activation-dependent antidepressant-relevant behavioral and synaptic actions of ketamine. J. Neurosci..

[92] Pothula S., Kato T., Liu R.J., Wu M., Gerhard D., Shinohara R., Sliby A.N., Chowdhury G.M.I., Behar K.L., Sanacora G. (2021). Cell-type specific modulation of NMDA receptors triggers antidepressant actions. Mol. Psychiatry.

[93] Moore T.J., Alami A., Alexander G.C., Mattison D.R. (2022). Safety and effectiveness of NMDA receptor antagonists for depression: A multidisciplinary review. Pharmacotherapy.

